# The Cost of Youth Suicide in Australia

**DOI:** 10.3390/ijerph15040672

**Published:** 2018-04-04

**Authors:** Irina Kinchin, Christopher M. Doran

**Affiliations:** 1 Centre for Indigenous Health Equity Research, School of Health, Medical and Applied Sciences, Central Queensland University, Brisbane 4000, Australia; c.doran@cqu.edu.au; 2 The Cairns Institute, James Cook University, Cairns 4870, Australia

**Keywords:** suicide, impact, cost, National Coronial Information System (NCIS), economics, adolescents

## Abstract

Suicide is the leading cause of death among Australians between 15 and 24 years of age. This study seeks to estimate the economic cost of youth suicide (15–24 years old) for Australia using 2014 as a reference year. The main outcome measure is monetized burden of youth suicide. Costs, in 2014 AU$, are measured and valued as direct costs, such as coronial inquiry, police, ambulance, and funeral expenses; indirect costs, such as lost economic productivity; and intangible costs, such as bereavement. In 2014, 307 young Australians lost their lives to suicide (82 females and 225 males). The average age at time of death was 20.4 years, representing an average loss of 62 years of life and close to 46 years of productive capacity. The average cost per youth suicide is valued at $2,884,426, including $9721 in direct costs, $2,788,245 as the value of lost productivity, and $86,460 as the cost of bereavement. The total economic loss of youth suicide in Australia is estimated at $22 billion a year (equivalent to US$ 17 billion), ranging from $20 to $25 billion. These findings can assist decision-makers understand the magnitude of adverse outcomes associated with youth suicide and the potential benefits to be achieved by investing in effective suicide prevention strategies.

## 1. Introduction

Suicide is a devastating global problem that occurs throughout the lifespan. It is estimated that over 800,000 people die from suicide each year, representing an annual global suicide rate of 10.7 per 100,000 population [1]. In Australia, the suicide rate is even higher, at 11.8 deaths per 100,000 [2,3]. In 2016 alone, 2862 Australians lost their lives to suicide, which is almost twice that of all transport-related fatalities (*n =* 1453) and is comparable to deaths from breast cancer (*n =* 3004) [4]. It is estimated that 370,000 Australians think about ending their life every year, 91,000 make a plan to suicide, and 65,000 suicide attempts occur each and every year [3]. The impact of suicide extends far and wide, with evidence that for every death by suicide, another six people are severely affected by intense grief that can continue for many years [5].

Of particular concern is the high level of suicidal behaviour among young people. Globally, suicide is the second most common cause of death among young people between 15 and 24 years of age [1]. In Australia, suicide is the leading cause of death in people aged between 15 and 24 [4]. Data from the Australian National Coronial Information System (NCIS), an online data repository of Australian coronial cases, suggest that between 2001 and 2014, 4460 young people in this age group died from suicide, equating to an average of 319 fatalities each year [2]. This is equivalent to a crude rate of youth suicide (15–24-year-olds) of 11.6 per 100,000, higher than countries with similar economic prosperity (as measured by gross domestic product per capita), including Canada (11.2 per 100,000), Hong Kong (10.3 per 100,000), the Netherlands (5.9 per 100,000), Sweden (8.9 per 100,000), and Singapore (6.7 per 100,000) [6].

Suicidal behaviour is often “the end result of the complex interaction between psychological, social, and biological factors” [7] and a more impulsive act in young people who are more likely to be influenced by media presentations of suicide and to die in cluster suicides [8,9]. Risks of suicidal behaviour increase in adolescence and young adulthood, particularly for the socially marginalized. The most prevalent risks include depression, alcohol abuse, mental disorders, antisocial behaviour, sexual abuse, physical abuse, poor peer relationships, suicidal behaviour by friends, family discord, family suicidal behaviour, unsupportive parents, living apart from parents, and social contagion [9].

In spite of the significant burden of harm associated with suicide, there is a dearth of information on its economic impact [10]. Most economic studies related to suicide and suicidal behaviour have concentrated on the impact for the wider population [11,12,13,14,15,16,17,18] or specific industries [19,20]. Only one study has considered the economic impact of youth suicide [21]. That study was undertaken in the U.S. and reported the economic cost of youth suicide to be $2.26 billion (in 1980 U.S. dollars). To the best of our knowledge, there has been no recent analysis eliciting the economic cost of youth suicide in Australia. These estimates can assist decision-makers to understand the magnitude of adverse outcomes associated with youth suicide and the potential benefits to be achieved by investing in effective strategies to address suicidal behaviour. The current study aims to quantify the economic cost of suicide among Australian youth (15–24 years old) using 2014 as a reference year.

## 2. Methods

The impact of youth suicide was assessed using: (1) years of life lost (YLL); (2) years of productive life lost (YPLL); and (3) the economic cost that considers direct, indirect, and intangible costs.

### 2.1. Suicide Data

A retrospective audit was performed of all closed cases from 1 January 2014 to 31 December 2014 stored in the NCIS. Data were extracted on the number of youth suicides by age (15–19 and 20–24), sex, means of suicide, and location of the incident [2].

### 2.2. Years of Life Lost (YLL)

The average age of a death by suicide (from NCIS) within each age group was subtracted from the average life expectancy at birth, 84.4 years for females and 80.3 years for males (Australian Bureau of Statistics (ABS) [22]) to obtain an estimate for YLL.

### 2.3. Years of Productive Life Lost (YPLL)

YPLL was derived by subtracting the average age of a death by suicide (from NCIS) within each age group from the retirement age in Australia (66 years) [23].

### 2.4. Economic Cost

A summary of the parameters associated with the economic cost of youth suicide is provided in Table 1.

#### 2.4.1. Direct Cost

Direct costs of a death by suicide included ambulance cost, coronial inquiry, policing costs, and funeral expenses. Unit cost and data sources are listed in Table 1. Not all costs were incurred in the event of every suicide. In the case of ambulance attendance, it was estimated that an ambulance only attended the scene in two-thirds of suicides [18].

#### 2.4.2. Indirect Cost

Lost economic productivity was calculated using the human capital method. This method considers the value of potential future earnings from time of incident to the retirement age assuming a discount profile and productivity loss. The discount profile considered gender-specific probability of survival to the retirement age, increased by an annual productivity factor of 1.75% [24], including the opportunity cost of saving (4.58%; [25]), discounted to present value using the rate of inflation (2.75%; [26]). Tax losses due to foregone income were valued at a tax rate of 25% [27]. The deadweight loss was measured as the value of taxation receipts foregone, equivalent to 28.75 cents in every foregone tax dollar [24].

Empirical estimates from the literature support the hypothesis that a lower incidence of mental illness is associated with higher income and, by implication, higher labour productivity. Psychological postmortem studies of suicides show that a psychiatric disorder is present at the time of death in most young people who die by suicide [28]. Accordingly, lost economic productivity estimates were adjusted to account for the impact of poor mental health on productivity potential and employment outcomes. Specifically, the average weekly earnings (AWEs) were reduced by 4.7% for males and by 3.1% for females [29]. A further assumption was made that 56% of those young people who died by suicide would have been employed and would otherwise have had the same expected productivity during the rest of their lives as other persons of the same age. This assumption is based on an employment rate of 65% (for those with no post-secondary education and no history of mental illness [30]) reduced by 9% probability of being employed (for those suffering from mental illness [31]).

#### 2.4.3. Intangible Cost

Bereavement cost was estimated using the unit cost of $14,410 per person incurred through lost productivity and health service utilisation [5] and the flow-on impact of grief that could affect up to six people [32].

### 2.5. Sensitivity Analysis

A number of univariate sensitivity analyses tested the robustness of the results to variations in key assumptions. Specifically, the number of suicides was decreased by 1%, 5%, and 10% (Sensitivity 1–3). The initial reduction in AWEs by 4.7% for males and 3.1% for females [29] was waived for both sexes (Sensitivity 4). A further assumption was made that 65% of youth would have been employed [30] instead of the baseline estimate of 56% (Sensitivity 5). The latter estimate was based upon an average probability of being employed with no post-secondary education and no history of mental illness [30]. Funeral cost was increased to $15,000 to reflect the upper limit of the average cost in Australia [33] (Sensitivity 6). The discount rate, which was used to convert future costs to present value (i.e., 4.58%), was adjusted to standardized rates of 4% and 5% (Sensitivity 7 and 8).

### 2.6. Ethical Approval

Ethics approval was granted by the Victorian Department of Justice (Project identification code: M0366) and the Central Queensland University Human Research Ethics Committees (H16/04-085).

## 3. Results

### 3.1. Youth Suicide Profile

Table 2 provides an overview of youth suicides in Australia for the year 2014. A total of 307 young Australians aged 15–24 died from suicide in 2014. The age-specific rate of youth suicide was 9.8 per 100,000 population. Regardless of the age cohort, the majority of suicides were males. Males aged 20–24 were more likely to die from suicide when compared to females (17.5 versus 6.2 per 100,000 population; *p* < 0.001) and their younger counterparts aged 15–19 years old (17.5 versus 10.1 per 100,000 population; *p* < 0.001). Threats to breathing were the most common cause of death (~75%) among all groups. On average, 64% of deaths occurred at home.

### 3.2. Economic Cost of a Single Youth Suicide

Table 3 displays the consequences of a single youth suicide by age and sex. The average number of years of life lost (YLL) was 62.1, and the average number of years of productive life lost (YPLL) was 45.6. These figures were somewhat higher for females than males and for 15 to 19 years old than 20 to 24 years old owing to the greater life span of the former subgroup. The loss of economic productivity attributable to each suicide averaged $2,788,245 in 2014 dollars, ranging from $2,041,139 for females aged 20–24 to $3,322,054 for males aged 15–19. The average cost of bereavement was estimated at $86,460. The direct economic cost per suicide was $9721.

### 3.3. Total Economic Cost of Youth Suicide 

As a consequence of youth suicide in 2014, the total YLL was estimated at 18,744 (Table 4). Of those lost years, 7869 (42%) would have been under the age of retirement (i.e., 66). The total economic cost of youth suicide in Australia was estimated at $21.97 billion (in 2014 AU$) per year, of which productivity loss was the largest cost accounting for 99.9%. The amount of economic productivity forgone was estimated at $21.94 billion. The cost of bereavement was the second largest cost of $26.54 million. Direct costs accounted for $2.98 million.

Table 5 considers the cost by means of suicide. Threat to breathing, including hanging, suffocation by putting a plastic bag or pillow over one’s head, and intentional drowning, accounted for more than 14,105 YLL and more than $16.53 billion of the total cost of suicide. Blunt force, such as jumping from a height, person moving in front of a moving object, such as a train or motor vehicle, and motor vehicle crash, was the second largest method of suicide among youth accounting for 2195 YLL and $2.58 billion. Piercing and penetrating force, including shooting with firearms and stabbing with a knife or other sharp instruments, claimed 907 YLL and $1.07 billion of the total cost of suicide in 2014.

### 3.4. Sensitivity Analysis

Table 6 provides results of the sensitivity analysis. The baseline cost of suicide of $21.97 billion ranged between $19.77 billion and $25.22 billion. The largest deviation related to the proportion of youth being employed (Sensitivity 5) and the theoretically reduced number of suicides per year (Sensitivity 1). For example, a reduction in youth suicides by 10% could potentially alleviate the economic burden by up to $2.20 billion per year.

## 4. Discussion

This research estimated the economic cost of suicide among Australian youth (15–24 years old) in 2014. To the best of our knowledge, this is the first study in Australia that translates youth suicide mortality data into economic terms. Our findings suggest that 18,744 years of life were lost as a result of suicide. Of those lost years, 7869 would have been under the age of retirement (i.e., 66). The economic cost of youth suicide in Australia is estimated at $21.97 billion a year ($19.77–$25.22 billion) in 2014 AU$ and equivalent to 5.5% (4.9–6.5%) of gross domestic product. Direct costs account for $2.98 million (0.01% of the total cost); indirect costs at $21.94 billion (99.87%); and intangible costs at $26.54 million (0.12%).

The cost of suicide increases by age cohort, from $8.86 billion (for 15–19-year-olds) to $13.07 billion (for 20–24-year-olds). This increase is attributable to the higher prevalence of suicide among 20–24-year-olds (12.0 per 100,000) compared to 15–19-year-olds (7.4 per 100,000). It is also worth noting that the cost of male youth suicide is significantly higher than the cost of female suicide ($17.51 billion versus $4.29 billion, respectively), again due to higher prevalence of suicide deaths in the former group (14.0 versus 5.4 per 100,000 population, respectively). Threats to breathing are the most common cause of death regardless of the age or gender and account for over $16.53 billion (75.24%) of the estimated total cost.

When discussing these key findings, it is important to reflect on the strengths and limitations of our approach. The best way of interpreting the costs in this paper is as a valuation of how much better off the society might be if there were no or less deaths from suicide, including a reduced risk of premature death, gained productivity and income, and more importantly reduced pain and suffering. All of these factors contribute to improved welfare or wellbeing and are amenable to monetary valuation. The costs of suicide thus correspond to a measure of the benefits to be secured if suicides were reduced.

This study has several strengths. The analysis utilises current suicide statistics from the best available evidence of suicide fatalities in Australia recorded in the NCIS. Suicide statistics is complex and “a particularly challenging cause to record and classify” [35]. The use of NCIS provides confidence in our estimates [36,37]. The costing methodology builds on our previous research and earlier studies in this area [10,14,20,21], but is also extended in several ways, in particular by focusing on youth from two age clusters, 15–19 and 20–24 years old. Unlike most suicide costing studies, our analysis factored in the postvention costs associated with bereavement and counselling [5]. Our method for estimating the cost of suicide is derived from an existing costing framework used by SafeWork Australia in estimating the cost of workplace injury and disease [24]. Key assumptions in our analysis and subsequent implications for results are tested using a range of sensitivity analyses.

Several limitations are also worth noting. By way of comparison with wider economic aggregates, we acknowledge that national income does not directly reflect human productivity loss and bereavement, but rather accounts for these costs indirectly through reduced output. The provisional nature of the human cost estimates should also be emphasised where the figures are based on assumptions, such as the probability of being employed and potential reduction in average weekly earnings. The coronial inquiry, police, and ambulance costs used in this analysis have been derived from published literature. However, these estimates are also derived using various assumptions that may impact on the ultimate accuracy of Australian-based values. Our analysis has made no attempt to estimate losses of quality of life to the victim’s family, friends, or others. Such an estimate would require survey data on psychological impacts and shall be pursued in future research. Further, according to Yang and Lester [38], cost savings might arise as a result of not having to treat the depressive and other psychiatric disorders of those who kill themselves; or avoidance of pension, social security, and nursing home care costs. Our costing methodology does not include these potential impacts.

The economic argument is an important tool for informing the development of and investment into evidence-informed suicide prevention strategies. Understanding the magnitude of costs of suicide can serve many purposes [39]. It can highlight the significant loss of productive capacity within a country and an estimate can be used to assess the potential benefits (or cost-savings) of implementing effective suicide prevention strategies to reduce youth suicide.

Reducing youth suicide requires a multifaceted approach. However, as pointed out by a number of systematic literature reviews, quality evidence of effective interventions for self-harming behaviours in young people is largely inadequate [40,41,42,43,44]. Given the magnitude of youth suicide, a new approach to suicide prevention is needed, with strong national direction backed by comprehensive, coordinated planning and implementation at a regional level [45]. This includes a better understanding of effective and cost-effective solutions to address this avoidable problem. A systems-based approach to suicide prevention in general was recently proposed in Australia that builds on nine strategies, including aftercare and crisis care; psychological and pharmacotherapy treatments; building the capacity and support of general practice teams; frontline staff training; gatekeeper training; school programs; community campaigns; media guidelines; and means restriction, which when implemented within a specific community at the same time are likely to lead to suicide reduction [46]. Although the effectiveness of this approach is yet to be established, our findings suggest that the impact of meeting a 10% reduction in youth suicide could potentially save many lives and over $2.20 billion each year.

## 5. Conclusions

Suicide is a leading cause of death in people between 15 and 24 years of age. An average of 319 young Australians take their lives each year at an economic cost of $22 billion. A better understanding of effective and cost-effective strategies is paramount to address this avoidable burden.

## Figures and Tables

**Table 1:** Summary of key cost parameters.

Cost Category	Unit Cost/Value (AU$/%)	Proportion of Suicides	Reference
*Direct cost*			
Funeral	4000	100%	Basic cremation cost in Australia [33]
Coronial	2595	100%	Per road fatality. Includes administrative costs, autopsy for 80% of deaths, and coronial inquests in 2% of deaths [14]
Ambulance	805	66%	Ambulance cost varies by state; the average cost of $805 was adopted (range $364–$1174). Based on 1.2 ambulances per attendance, and attendance of at least one ambulance at 54% of all deaths by suicide [18]
Police	2595	100%	Based on New Zealand (NZ) police costs adjusted to 2014 $ [14]
*Indirect cost*			
Productivity loss:			
Average weekly earnings (AWEs)	$940 ^1^ (Male) $1430 ^1^ (Female) $1182 ^1^ (Person)	56%	Proxy for productivity, Australian Bureau of Statistics (ABS) 6306.0 Employee Earnings and Hours survey, May 2014. Weighted AWEs by sex [34]
Discount rate	4.58%	56%	Opportunity cost of money: Average of rates of return for private and government saving instruments and Reserve Bank of Australia target for March 2005 to December 2014 [25]
Inflation rate	2.75%	56%	Average of annual weighted ABS 6401.0 Consumer Price Index from December 2004 to December 2014 [26]
Productivity rate	1.75%	56%	Annual increase in productivity. Safe Work Australia report 2012–2013 [24]
Average tax for foregone earnings	25.00%	56%	Australian Taxation Office [27]
Transfer costs	28.75%	56%	Deadweight cost of tax losses. Safe Work Australia report 2012–2013 [24]
*Intangible cost*			
Bereavement/Postvention	$14,410	600%	Corso, Mercy, Simon, Finkelstein, and Miller [32] estimated that for each suicide up to six people are affected, incurring a cost to society of $14,410 per person as valued by Comans, Visser, and Scuffham [5]

^1^ Unadjusted; reduced by 4.7% for males, by 3.1% for females in baseline analysis, and varied in sensitivity analysis.

**Table 2:** Profile of youth suicide (15–24 years old) in Australia, 2014.

Variable	Age 15–19			Age 20–24			Age 15–24		
	Female	Male	Persons	Female	Male	Persons	Female	Male	Persons
Number of suicides (%)	32 (29%)	77 (71%)	109 (100%)	50 (25%)	148 (75%)	198 (100%)	82 (27%)	225 (73%)	307 (100%)
Age-specific rate (per 100,000 population)	4.5	10.1	7.4	6.2	17.5	12.0	5.4	14.0	9.8
Mean age of suicide	17.0	17.4	17.3	22.3	22.1	22.2	20.3	20.5	20.4
Cause of death									
Threat to breathing	26 (81%)	60 (78%)	86 (79%)	36 (72%)	109 (74%)	145 (73%)	62 (76%)	169 (75%)	231 (75%)
Blunt force	3 (9%)	10 (13%)	13 (12%)	6 (12%)	17 (11%)	23 (12%)	9 (11%)	27 (12%)	36 (12%)
Poisoning	2 (6%)	1 (1%)	3 (3%)	7 (14%)	12 (8%)	19 (10%)	9 (11%)	13 (6%)	22 (7%)
Piercing, penetrating force	1 (3%)	4 (5%)	5 (5%)	1 (2%)	9 (6%)	10 (5%)	2 (2%)	13 (6%)	15 (5%)
Other	-	2 (3%)	2 (2%)	-	1 (1%)	1 (1%)	-	3 (1%)	3 (1%)
Suicide (incident) location									
Home	20 (63%)	48 (62%)	68 (62%)	35 (70%)	95 (64%)	130 (66%)	55 (67%)	143 (64%)	198 (64%)
Public area (incl. transport)	11 (34%)	21 (27%)	32 (29%)	13 (26%)	46 (31%)	59 (30%)	24 (29%)	67 (30%)	91 (30%)
Other	1 (3%)	8 (10%)	9 (8%)	2 (4%)	7 (5%)	9 (5%)	3 (4%)	15 (7%)	18 (6%)

**Table 3:** The economic cost of a single youth suicide, Australia, 2014, by age and sex.

Variable	Years of Life Lost	Year of Productive Life Lost	Economic Cost (AU$)			Total
			Direct	Indirect	Intangible	
Age 15–19						
Female	67.5	49.0	$9721	$2,156,865	$86,460	$2,253,046
Male	63.0	48.6	$9721	$3,225,873	$86,460	$3,322,054
Person	65.2	48.7	$9721	$2,962,760	$86,460	$3,058,941
Age 20–24						
Female	62.2	43.7	$9721	$1,944,958	$86,460	$2,041,139
Male	58.3	43.9	$9721	$2,908,937	$86,460	$3,005,118
Person	60.2	43.8	$9721	$2,671,674	$86,460	$2,767,855
Age 15–24						
Female	64.2	45.7	$9721	$2,029,820	$86,460	$2,126,001
Male	59.9	45.5	$9721	$3,035,860	$86,460	$3,132,041
Persons	62.1	45.6	$9721	$2,788,245	$86,460	$2,884,426

**Table 4:** The total economic cost of youth suicide, Australia, 2014, by age and sex.

Variable	Years of Life Lost	Year of Productive Life Lost	Economic Cost (AU$)			Total
			Direct	Indirect	Intangible	
Age 15–19						
Female	2159	882	$311,057	$1,901,480,033	$2,766,720	$1,904,557,811
Male	4849	2104	$748,482	$6,787,636,004	$6,657,420	$6,795,041,906
Person	7008	2986	$1,059,539	$8,845,965,126	$9,424,140	$8,856,448,805
Age 20–24						
Female	3108	1228	$486,027	$2,388,710,944	$4,323,000	$2,393,519,971
Male	8628	3655	$1,438,641	$10,632,781,756	$12,796,080	$10,647,016,476
Person	11,736	4883	$1,924,668	$13,046,768,339	$17,119,080	$13,065,812,087
Age 15–24						
Female	5267	2110	$797,085	$4,282,413,273	$7,089,720	$4,290,300,078
Male	13,477	5759	$2,187,123	$17,484,538,814	$19,453,500	$17,506,179,437
Persons	18,744	7869	$2,984,208	$21,940,940,814	$26,543,220	$21,970,468,242

**Table 5:** The total economic cost of youth suicide, Australia, 2014, by means of suicide.

Variable	Years of Life Lost	Year of Productive Life Lost	Economic Cost (AU$)			Total
			Direct	Indirect	Intangible	
Threat to breathing	14,105	5921	$2,245,446	$16,509,307,258	$19,972,260	$16,531,524,964
Blunt force	2195	923	$349,940	$2,572,879,053	$3,112,560	$2,576,341,553
Exposure to chemical or other substance	1357	564	$213,852	$1,572,314,977	$1,902,120	$1,574,430,949
Piercing, penetrating force	907	384	$145,808	$1,072,032,939	$1,296,900	$1,073,475,647
Other	180	77	$29,162	$214,406,588	$259,380	$214,695,129
Total	18,744	7869	$2,984,207	$21,940,940,814	$26,543,220	$21,970,468,242

**Table 6:** Sensitivity analysis of key parameters (in AU$).

Parameter Varied	Total Cost of Youth Suicide (AU$)
Number of suicides	
Sensitivity 1 = 276 (reduction by 10%)	$19,773,421,418
Sensitivity 2 = 292 (reduction by 5%)	$20,871,944,830
Sensitivity 3 = 304 (reduction by 1%)	$21,750,763,559
Baseline = 307	$21,970,468,242
Average weekly earnings	
Baseline = $911 (Female); $1363 (Male); $1251 (Person)	$21,970,468,242
Sensitivity 4 = $940 (Female); $1430 (Male); $1302 (Person)	$22,860,891,438
Proportion of youth that employed	
Baseline = 56%	$21,970,468,242
Sensitivity 5 = 65%	$25,223,000,172
Funeral cost	
Baseline = 4000	$21,970,468,242
Sensitivity 6 = 15,000	$21,973,845,242
Discount rate used to convert future costs to present value	
Sensitivity 7 = 4%	$25,125,243,025
Baseline = 4.58%	$21,970,468,242
Sensitivity 8 = 5%	$20,046,012,345

## References

[1] World Health Organization (2014). Preventing Suicide: A Global Imperative.

[2] National Coronial Information System (NCIS). http://www.ncis.org.au/.

[3] Australian Bureau of Statistics (2007). National Survey of Mental Health and Wellbeing.

[4] Australian Bureau of Statistics (2017). Underlying Cause of Death in Australia.

[5] Comans T., Visser V., Scuffham P. (2013). Cost Effectiveness of a Community-Based Crisis Intervention Program for People Bereaved by Suicide. Crisis.

[6] World Health Organisation (2016). WHO Mortality Database.

[7] De Leo D., Burgis S., Bertolote J.M., Kerkhof A.J., Bille-Brahe U. (2006). Definitions of Suicidal Behavior: Lessons Learned from the Who/Euro Multicentre Study. Crisis.

[8] Patton G.C. (2014). Youth Suicide: New Angles on an Old Problem. J. Adolesc. Health.

[9] Bridge A.J., Goldstein T.R., Brent D.A. (2006). Adolescent Suicide and Suicidal Behavior. J. Child Psychol. Psychiatry.

[10] Kinchin I., Doran C.M., Hall W.D., Meurk C. (2017). Understanding the True Economic Impact of Self-Harming Behaviour. Lancet Psychiatry.

[11] SMARTRISK (2009). The Economic Burden of Injury in Canada.

[12] Shepard D.S., Gurewich D., Lwin A.K., Reed G.A., Silverman M.M. (2016). Suicide and Suicidal Attempts in the United States: Costs and Policy Implications. Suicide Life Threat Behav..

[13] O’Dea D., Tucker S. (2005). The Cost of Suicide to Society.

[14] KPMG International (2013). Economic Cost of Suicide in Australia.

[15] Kinder A., Cooper C.L. (2009). The Costs of Suicide and Sudden Death within an Organization. Death Stud..

[16] Kennelly B. (2007). The Economic Cost of Suicide in Ireland. Crisis.

[17] ConNetica Consulting (2009). The Estimation of the Economic Cost of Suicide to Australia.

[18] Clayton D., Barcelo A. (1999). The Cost of Suicide Mortality in New Brunswick, 1996. Chronic Dis. Can..

[19] Christopher M.D., Ling R., Milner A., Kinchin I. (2016). The Economic Cost of Suicide and Non-Fatal Suicidal Behaviour in the Australian Construction Industry. Int. J. Ment. Health Psychiatry.

[20] Kinchin I., Doran C.M. (2017). The Economic Cost of Suicide and Non-Fatal Suicide Behavior in the Australian Workforce and the Potential Impact of a Workplace Suicide Prevention Strategy. Int. J. Environ. Res. Public Health.

[21] Weinstein M.C., Saturno P.J. (1989). Economic Impact of Youth Suicide and Suicide Attempts. Report of the Secretary’s Task Force on Youth Suicide.

[22] Australian Bureau of Statistics (2016). 3302.0.55.001—Life Tables, States, Territories and Australia, 2013–2015.

[23] Department of Social Services Age Pension, Australian Government. https://www.dss.gov.au/seniors/benefits-payments/age-pension.

[24] Safe Work Australia (2015). The Cost of Work-Related Injury and Illness for Australian Employers, Workers, and the Community, 2012–13.

[25] Reserve Bank of Australia (RBA) Cash Rate. http://www.rba.gov.au/statistics/cash-rate/.

[26] Australian Bureau of Statistics (2016). 6401.0—Consumer Price Index, Australia, Sep 2016.

[27] Australian Taxation Office Simple Tax Calculator. https://www.ato.gov.au/Calculators-and-tools/Simple-tax-calculator/.

[28] Osafo J., Hjelmeland H., Akotia C.S., Knizek B.L. (2011). The Meanings of Suicidal Behaviour to Psychology Students in Ghana: A Qualitative Approach. Transcult. Psychiatry.

[29] Forbes M., Barker A., Turner S. (2010). The Effects of Education and Health on Wages and Productivity. Productivity Commission Staff Working Paper.

[30] Committee for Economic Development of Australia (2015). Australia’s Future Workforce?.

[31] Sainsbury Centre for Mental Health (2003). The Economic and Social Cost of Mental Illness.

[32] Corso P.S., Mercy J.A., Simon T.R., Finkelstein E.A., Miller T.R. (2007). Medical Costs and Productivity Losses Due to Interpersonal and Self-Directed Violence in the United States. Am. J. Prev. Med..

[33] Australian Security and Investments Commission Paying for Your Funeral. https://www.moneysmart.gov.au/life-events-and-you/over-55s/paying-for-your-funeral.

[34] Australian Bureau of Statistics (2014). 6306.0—Employee Earnings and Hours Survey, May 2014.

[35] Bradley C.E., Harrison J.E., Elnour A.A. (2010). Appearances May Deceive: What’s Going on with Australian Suicide Statistics?. Med. J. Aust..

[36] Bugeja L., Clapperton A.J., Killian J.J., Stephan K.L., Ozanne-Smith J. (2010). Reliability of Icd-10 External Cause of Death Codes in the National Coroners Information System. HIM J..

[37] Daking L., Dodds L. (2007). Icd-10 Mortality Coding and the NCIS: A Comparative Study. HIM J..

[38] Yang B., Lester D. (2007). Recalculating the Economic Cost of Suicide. Death Stud..

[39] McDavid D., O’Connor R.C., Pirkis J. (2016). Making an Economic Case for Investing in Suicide Prevention. The International Handbook of Suicide Prevention.

[40] Robinson J., Cox G., Malone A., Williamson M., Baldwin G., Fletcher K., O’Brien M. (2013). A Systematic Review of School-Based Interventions Aimed at Preventing, Treating, and Responding to Suicide-Related Behavior in Young People. Crisis.

[41] Robinson J., Calear A.L., Bailey E. (2018). Suicide Prevention in Educational Settings: A Review. Australas. Psychiatry.

[42] Zalsman G., Hawton K., Wasserman D., van Heeringen K., Arensman E., Sarchiapone M., Carli V., Höschl C., Barzilay R., Balazs J. (2016). Suicide Prevention Strategies Revisited: 10-Year Systematic Review. Lancet Psychiatry.

[43] Harrod C.S., Goss C.W., Stallones L., DiGuiseppi C. (2014). Interventions for primary prevention of suicide in university and other post-secondary educational settings. Cochrane Database Syst. Rev..

[44] Bustamante M.L., Eddleston M., Hansen K.S., Konradsen F. (2018). Quality Assessment of Economic Evaluations of Suicide and Self-Harm Interventions. Crisis.

[45] Suicide Prevention Australia (2014). Discussion Paper: One World Connected: An Assessment of Australia’s Progress in Suicide Prevention.

[46] Black Dog Institute (2016). An Evidence Based Systems Approach to Suicide Prevention: Guidance on Planning, Commissioning and Monitoring.

