# Correction: Kinchin, I.; Doran, C.M. The Cost of Youth Suicide in Australia. *Int. J. Environ. Res. Public Health* 2018, *15*, 672

**DOI:** 10.3390/ijerph15091940

**Published:** 2018-09-06

**Authors:** Irina Kinchin, Christopher M. Doran

**Affiliations:** 1 Centre for Indigenous Health Equity Research, School of Health, Medical and Applied Sciences, Central Queensland University, Brisbane 4000, Australia; c.doran@cqu.edu.au; 2 The Cairns Institute, James Cook University, Cairns 4870, Australia

The authors wish to add the following corrections to their paper published in the International Journal of Environmental Research and Public Health [1]. When computing Total Indirect Cost column in Table 4, Indirect Cost per single youth suicide was multiplied by Total Years of Productive Life Lost (YPLL). The correct calculation should have been Indirect Cost per single youth suicide multiplied by Total Number of Suicides (not YPLL). This led to an overestimated Total Indirect Cost and Total Cost of Youth Suicide in Australia reported in the paper. Following further suggestion by a reviewer, we aligned the wording under Section 3.2 and Section 3.3 (i.e., Productivity Loss and Bereavement) with the terms used in tables (i.e., Indirect Cost and Intangible Cost).

In the abstract, the sentence regarding the Total Cost should be as follows: The total economic loss of youth suicide in Australia is estimated at $511 million a year (equivalent to US$ 352 million), ranging from $460 to $586 million. Abstract should read as follows.

 

Abstract: Suicide is the leading cause of death among Australians between 15 and 24 years of age. This study seeks to estimate the economic cost of youth suicide (15–24 years old) for Australia using 2014 as a reference year. The main outcome measure is monetized burden of youth suicide. Costs, in 2014 AU$, are measured and valued as direct costs, such as coronial inquiry, police, ambulance, and funeral expenses; indirect costs, such as lost economic productivity; and intangible costs, such as bereavement. In 2014, 307 young Australians lost their lives to suicide (82 females and 225 males). The average age at time of death was 20.4 years, representing an average loss of 62 years of life and close to 46 years of productive capacity. The average cost per youth suicide is valued at $2,884,426, including $9721 in direct costs, $2,788,245 as the value of lost productivity, and $86,460 as the cost of bereavement. The total economic loss of youth suicide in Australia is estimated at $511 million a year (equivalent to US$352 million), ranging from $460 to $586 million. These findings can assist decision-makers understand the magnitude of adverse outcomes associated with youth suicide and the potential benefits to be achieved by investing in effective suicide prevention strategies.

 

Results should read as follows:

## 3. Results

### 3.2. Economic Cost of a Single Youth Suicide

Table 3 displays the consequences of a single youth suicide by age and sex. The indirect cost as the loss of economic productivity attributable to each suicide averaged $2.8 million in 2014 dollars, ranging from $1.9 million for females aged 20–24 to $3.2 million for males aged 15–19. The intangible cost represented by the average cost of bereavement was estimated at $86,460. The direct economic cost per suicide was $9721.

### 3.3. Total Economic Cost of Youth Suicide 

The total economic cost of youth suicide in Australia was estimated at $511.1 million (in 2014 AU$) per year, of which the indirect cost was the largest cost accounting for 94% (Table 4). The amount of indirect costs was estimated at $481.6 million. The cost of bereavement (intangible cost) was the second largest cost of $26.5 million. Direct costs accounted for just under $3 million.

Table 5 considers the cost by means of suicide. Threat to breathing, including hanging, suffocation by putting a plastic bag or pillow over one’s head, and intentional drowning, accounted for more than 14,105 YLL and more than $384.6 million of the total cost of suicide. Blunt force, such as jumping from a height, person moving in front of a moving object, such as a train or motor vehicle, and motor vehicle crash, was the second largest method of suicide among youth accounting for 2195 YLL and $59.9 million. Piercing and penetrating force, including shooting with firearms and stabbing with a knife or other sharp instruments, claimed 907 YLL and just under $25 million of the total cost of suicide in 2014.

### 3.4. Sensitivity Analysis

Table 6 provides results of the sensitivity analysis. The baseline cost of suicide of $511.1 million ranged between $460.0 and $585.9 million. The largest deviation related to the proportion of youth being employed (Sensitivity 5) and the theoretically reduced number of suicides per year (Sensitivity 1). For example, a reduction in youth suicides by 10% could potentially alleviate the economic burden by up to $51.1 million per year including $298,421 ($0.3 million) in direct costs, $2.7 million as intangible, and $48.2 million as indirect costs.

## 4. Discussion

The economic cost of youth suicide in Australia is estimated at $511 million a year ($460–586 million) in 2014 AU$. Direct costs account for $3 million (1% of the total cost); indirect costs at $482 million (94%); and intangible costs at $27 million (5%).

The cost of suicide increases by age cohort, from $192 million (for 15–19-year-olds) to $317 million (for 20–24-year-olds). This increase is attributable to the higher prevalence of suicide among 20–24-year-olds (12.0 per 100,000) compared to 15–19-year-olds (7.4 per 100,000). It is also worth noting that the cost of male youth suicide is significantly higher than the cost of female suicide ($406 million versus $102 million, respectively), again due to higher prevalence of suicide deaths in the former group (14.0 versus 5.4 per 100,000 population, respectively). Threats to breathing are the most common cause of death regardless of the age or gender and account for over $385 million (75%) of the estimated total cost.

Although the effectiveness of this approach is yet to be established, our findings suggest that the impact of meeting a 10% reduction in youth suicide could potentially save many lives and over $51 million a year, including $298,421 ($0.3 million) in direct costs, $3 million as intangible and $48 million as indirect costs.

## 5. Conclusions

An average of 319 young Australians take their lives each year at an economic cost of $511 million. 

## Other Corrections:

In Table 1, the average weekly earnings for females were $940 and for males—$1430.

Reference 19 should read:

19 Doran, C.M.; Ling, R.; Milner, A.; Kinchin, I. The Economic Cost of Suicide and Non-Fatal Suicidal Behaviour in the Australian Construction Industry. *Int. J. Ment. Health Psychiatry* **2016**, *2*, doi:10.4172/2471-4372.1000130.

## Figures and Tables

**Table 3:** The economic cost of a single youth suicide, Australia, 2014, by age and sex.

Variable	Years of Life Lost	Year of Productive Life Lost	Economic Cost (AU$)			Total
			Direct	Indirect	Intangible	
Age 15–19						
Female	67.5	49.0	$9721	$2,156,865	$86,460	$2,253,046
Male	63.0	48.6	$9721	$3,225,873	$86,460	$3,322,054
Person	65.2	48.7	$9721	$2,962,760	$86,460	$3,058,941
Age 20–24						
Female	62.2	43.7	$9721	$1,944,958	$86,460	$2,041,139
Male	58.3	43.9	$9721	$2,908,937	$86,460	$3,005,118
Person	60.2	43.8	$9721	$2,671,674	$86,460	$2,767,855
Age 15–24						
Female	64.2	45.7	$9721	$2,029,820	$86,460	$2,126,001
Male	59.9	45.5	$9721	$3,035,860	$86,460	$3,132,041
Persons	62.1	45.6	$9721	$2,788,245	$86,460	$2,884,426

**Table 4:** The total economic cost of youth suicide, Australia, 2014, by age and sex.

Variable	Years of Life Lost ^1^	Year of Productive Life Lost ^1^	Economic Cost (AU$)			Total
			Direct	Indirect	Intangible	
Age 15–19						
Female	2159	882	$311,057	$38,830,479	$2,766,720	$41,908,256
Male	4849	2104	$748,482	$139,745,447	$6,657,420	$147,151,349
Person	7008	2986	$1,059,539	$181,686,489	$9,424,140	$192,170,168
Age 20–24						
Female	3108	1228	$486,027	$54,711,657	$4,323,000	$59,520,684
Male	8628	3655	$1,438,641	$242,212,052	$12,796,080	$256,446,773
Person	11,736	4883	$1,924,668	$297,610,614	$17,119,080	$316,654,362
Age 15–24						
Female	5267	2110	$797,084	$93,642,104	$7,089,720	$101,528,908
Male	13,477	5759	$2,187,123	$384,294,347	$19,453,500	$405,934,970
Persons	18,744	7869	$2,984,207	$481,580,670	$26,543,220	$511,108,097

^1^ Totals might deviate due to rounding.

**Table 5:** The total economic cost of youth suicide, Australia, 2014, by means of suicide.

Variable	Years of Life Lost	Year of Productive Life Lost	Economic Cost (AU$)			Total
			Direct	Indirect	Intangible	
Threat to breathing	14,105	5921	$2,245,446	$362,362,002	$19,972,260	$384,579,708
Blunt force	2195	923	$349,940	$56,472,000	$3,112,560	$59,934,500
Exposure to chemical or other substance	1357	564	$213,852	$34,510,667	$1,902,120	$36,626,639
Piercing, penetrating force	907	384	$145,808	$23,530,000	$1,296,900	$24,972,708
Other	180	77	$29,162	$4,706,000	$259,380	$4,994,542
Total	18,744	7869	$2,984,207	$481,580,670	$26,543,220	$511,108,097

**Table 6:** Sensitivity analysis of key parameters (in AU$).

Parameter Varied	Total Cost of Youth Suicide (AU$)
Number of suicides	
Sensitivity 1 = 276 (reduction by 10%)	$459,997,288
Sensitivity 2 = 292 (reduction by 5%)	$485,552,692
Sensitivity 3 = 304 (reduction by 1%)	$505,997,016
Baseline = 307	$511,108,097
Average weekly earnings	
Baseline = $911 (Female); $1363 (Male); $1251 (Person)	$511,108,097
Sensitivity 4 = $940 (Female); $1430 (Male); $1302 (Person)	$530,651,954
Proportion of youth that employed	
Baseline = 56%	$511,108,097
Sensitivity 5 = 65%	$585,921,731
Funeral cost	
Baseline = 4000	$511,108,097
Sensitivity 6 = 15,000	$514,485,097
Discount rate used to convert future costs to present value	
Sensitivity 7 = 4%	$580,352,099
Baseline = 4.58%	$511,108,097
Sensitivity 8 = 5%	$468,868,306
